# The assessment and treatment of the musculoskeletal manifestations of cystinosis

**DOI:** 10.3389/fneph.2025.1624586

**Published:** 2025-09-25

**Authors:** Priya Singh, D’Arcy Marsh, Melinda Sharkey

**Affiliations:** Department of Orthopaedic Surgery, Montefiore Health System, Bronx, NY, United States

**Keywords:** scoliosis, myopathy, low bone mineral density, fragility fractures, bone deformity, renal osteodystrophy, long bone fractures, vertebral insufficiency fractures

## Abstract

Cystinosis is a rare autosomal recessive lysosomal storage disease caused by a defective lysosomal cystine carrier protein, cystinosin, resulting in formation and deposition of cystine crystals throughout the body. The renal manifestations of the disease have long been studied, but the musculoskeletal consequences of the disease are generally less well understood. Limb deformities, scoliosis, myopathy and low bone mineral density are associated with cystinosis and can lead to pain, fragility fractures, bone deformity, and difficulty ambulating. Although potentially exacerbated by renal disease and post-transplant medications, it has been found that the musculoskeletal manifestations of cystinosis are also due to inherent dysfunction caused by the mutation of cystinosin. Surgical intervention can provide solutions to the bony symptoms of cystinosis. Early referral to an orthopedic surgeon and evaluation for corrective scoliosis surgery, guided growth for growing children with lower extremity deformity and formal osteotomies for deformity correction in skeletally mature individuals may improve physical function and decrease pain. Standard principles of operative treatment of scoliosis and of bone deformity correction utilized for the treatment of bone deformity in other metabolic bone disease may be applied to patients with cystinosis in the absence of cystinosis-specific studies of the efficacy and outcomes of orthopedic surgery.

## Introduction

Cystinosis, which often presents as nephropathic cystinosis in infants, is known to cause cystine crystal accumulation in cells of all tissues; however, the damage is heterogeneous and can vary in both severity and rate of progression (1). The progression to Fanconi syndrome and chronic kidney disease leads to the failure of renal tubules to reabsorb small molecules and results in wasting of important inorganic components important to bone health including calcium and phosphate. This leads to poor bone mineralization, rickets and osteomalacia. Additionally, it has been found that normal cystinosin is required for healthy osteoclast activity, suggesting a direct effect of this mutation beyond that of electrolyte wasting on bone biology (2).

As the life expectancy of children with cystinosis increases with the development of disease modifying drugs like cysteamine, the associated skeletal deformities and other orthopedic manifestations of this disease may become more consequential. The skeletal consequences of cystinosis have been termed Cystinosis Metabolic Bone Disease (CMBD), and include long bone deformity, scoliosis, vertebral fragility fractures, low bone mass, renal osteodystrophy, and a possibly increased risk of long bone fractures (3). Long bone deformity causes pain and difficulty ambulating; compensatory mechanisms for an altered gait results in sequela that can include back and hip pain. Vertebral insufficiency fractures may cause debilitating back pain. Case studies have shown that guided growth procedures can be used to correct long bone deformities and posterior spinal fusions can treat scoliosis (1, 4).

Overall, the impact of cystinosis on bone and muscle is complex and multifactorial, and the consequences can have a myriad of effects on patients’ quality of life. It is important for these patients to receive comprehensive care for CMBD, including early referral to a pediatric orthopedic deformity specialist. This review provides a comprehensive summary of the current knowledge of the musculoskeletal manifestations of cystinosis, particularly in children and adolescents, and surgical treatment options for lower limb and spine deformity.

## Musculoskeletal clinical manifestations of cystinosis

As children with cystinosis age, they develop the constellation of skeletal complications known as CMBD: bone deformity, decreased bone mass, and incidental vertebral fractures.

The destruction of renal proximal tubules by cysteine crystal deposition leads to urinary loss of calcium, phosphate and vitamin D binding protein, as well as the decreased activity of alpha-1 hydroxylase (3). Clinically, children with CMBD may present with short stature, rickets and limb deformities. Eventually, patients may progress to renal osteodystrophy and osteomalacia (5). Patients with cystinosis have been found to have both genu varum (“bowed legs”) and genu valgum (“knocked knees”) (1, 3, 4). These deformities are often noted around early adolescence (4) and have been associated with walking impairment (3).

A study of 30 patients with nephropathic cystinosis treated at the NIH showed remarkably high rates of musculoskeletal issues. Sixty-four percent of patients had long bone deformity, 50% had scoliosis, 46% had low bone mass, 32% had incidental vertebral fractures, and 27% had already suffered at least one long bone fracture. Of note, 21% had renal osteodystrophy (all of whom had undergone transplant) (1).

Interestingly, the decreased bone mass experienced by these patients may not only be due to malabsorption secondary to renal dysfunction, but also due to intrinsic mutations affecting osteogenic cells. Several *in vitro* studies show that the CTNS mutations affecting cystinosin are correlated with osteoclast and osteoblast dysfunction leading to reduced bone remodeling activity (3, 6–8). Cysteamine applied to osteoclasts isolated from peripheral blood had no impact on osteoclastogenesis in healthy individuals, but impaired *in vitro* osteoclastic resorption at high doses (200µM) and stimulated osteoblastic differentiation and maturation in low doses (50µM). The decreased osteoblastic proliferation caused by higher doses of cysteamine has been shown to have an inhibitory effect on bone mineralization (2, 7, 9). Of note, mutated mice in these studies did not show signs of renal Fanconi syndrome, thereby supporting the conclusion that the cellular dysfunction may not be caused by renal dysfunction but rather intrinsic mutations (7). Furthermore, Florezano et al. found that their patients experienced skeletal deformities, decreased bone mass, and insufficiency fractures prior to the onset of end stage renal disease (1). Other studies have corroborated that bone mineral density is not correlated with transplant status either (9).

Although renal transplant has become a mainstay of treatment for patients that have progressed to end stage renal disease, transplantation and the immunosuppressive medications required may worsen musculoskeletal symptoms. Glucocorticoids may limit further growth, contributing to short stature, and cause osteoporosis and increased fracture risk (9). Likewise, approximately a third of patients with cystinosis experience insufficiency fractures of the long bones or vertebrae (1–3, 10, 11). The 27% of children with cystinosis who experience a fracture may have suffered lower energy injuries that would not harm healthy children (1). It is important to acknowledge, however, that a large prospective cohort study of over 500 children with chronic kidney disease developed fractures at a rate of 2.4- to 3-fold higher than the general population, so it is possible that the smaller studies that exist on children with cystinosis underestimate the rate of incident fractures (12). Vertebral insufficiency fractures are often found incidentally in patients prior to end stage renal disease or renal transplant and have a predilection for the lumbar spine (1).

Cystinotic myopathy is another common manifestation of this disease impacting muscle, bone and joint development (3, 13, 14). As children grow, the mechanical stimulation of muscle activity at their attachments to the long bones induces bone growth and mineralization. Therefore, if musculature is weakened, as has been proven molecularly with lower levels of muscle and plasma carnitine in CMBD (3, 15, 16), it can worsen the already lower bone mineral density. Without appropriate medical treatment of the disease, this will progress to general muscle weakness and has even been associated with respiratory muscle weakness (17). This can potentially be treated and prevented by regular physical therapy and exercise focused on muscle strengthening.

## Orthopedic surgical management

### Initial workup

**Figure 1:**
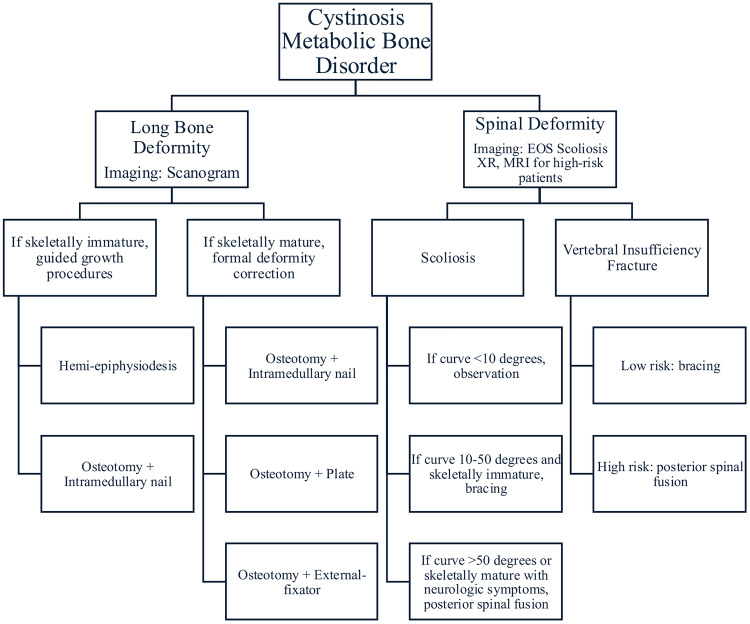
This is the general treatment algorithm with which to approach cystinosis. This is not a comprehensive chart as much of treatment is individualized, however it provides an overview of available treatment options.

Once concern for musculoskeletal manifestations of cystinosis has arisen, further diagnostic imaging should be obtained to guide the next steps in management. If clinical concern for scoliosis is present, upright full spine radiographs should be obtained (3). The best imaging to assess leg length and deformity is a scanogram, which is obtained either by a single radiograph taken on one long plate of the bilateral legs from hip to feet (teleroentgenogram) or three images taken of the ankles, knees, and hips that are stitched together electronically (orthoroentgenogram). The accessibility of EOS (Edge-On Scanning) machines, which use low-dose radiation to take both anterior-posterior (AP) and lateral radiographs simultaneously, provides improved information on length of rotation of the spine and limbs (**Figure 1**). This has improved the utility of two-dimensional imaging in limb deformity. Angular deformities can then be measured using a PACS application. Radiographs of the hand can reveal information on the patient’s bone age, and radiographs of the wrist and knee can be evaluated for widening, fraying or cupping of the metaphysis or fragmentation of the physis, which would be concerning for rickets.

Although it is important to monitor bone density in patients with cystinosis, dual-energy X-ray absorptiometry (DEXA) scans are not usually indicated in pediatric patients with CKD (3, 9, 18). DEXA scans are based on the area not volume of bone, which results in an underestimation of bone density in smaller patients. Studies have shown at best a modest reliability of DEXA to predict trabecular BMD or cortical bone mineral content (BMC) (19). The 2017 Kidney Disease: Improving Global Outcomes (KDIGO) clinical practice guidelines do not recommend obtaining a DEXA scan for pediatric CKD-MBD patients, as no evidence has been established regarding ability of DEXA scans to predict fracture risk in pediatric patients (20). DEXA scans were not discussed in the 2024 edition of the KDIGO clinical practice guidelines.

Magnetic resonance imaging (MRIs) are not routinely obtained for patients that have scoliosis or vertebral insufficiency fractures. They are recommended for patients that experience any neurologic symptoms, abnormal abdominal reflexes, have a left sided thoracic major curve, or present with scoliosis before the age of 10 years old. MRIs provide more detail about the spinal cord, nerve roots and soft tissue structures. They can help identify neural axis abnormalities like tethered cord syndromes, syrinxes and Chiari malformations. Children with cystinosis have a 12-fold higher prevalence of Chiari malformations (21). Therefore, there should be a low threshold to obtain MRI brain and full spine for cystinosis patients that present with new-onset neurologic symptoms, headache, ataxia, or incontinence.

Laboratory values should also be obtained as part of the initial workup to determine the need for nutritional supplementation. In particular, calcium, vitamin D, phosphate, alkaline phosphatase and parathyroid hormone levels should be routine monitored and repleted with calcium, phosphate, cholecalciferol, and calcitriol supplementation as necessary. However, supplementation is not without its inherent risks, as it has been associated with nephrocalcinosis and nephrolithiasis due to the increased urinary renal calcium wasting these patient experience (1). Additional tests include hormone levels, specifically luteinizing hormone, follicular stimulating hormone and total testosterone, as these patients can experience hypogonadism and hormonal repletion may also improve bone mineral density.

No standardized surgical treatment protocol has been designed specifically for children with cystinosis due to the rarity of the disease. Medical management targeted at minimizing manifestations of cystinosis can be implemented from the time of diagnosis. When caring for these children, it’s important to routinely examine for scoliosis and lower limb deformity. As children grow, scoliosis and long bone deformities have the potential to progress, so it is essential these patients are referred to a pediatric orthopedic surgeon as early as possible for evaluation and potential intervention.

### Long bone deformity

To better explain the surgical options for lower limb deformity correction, an understanding of the normal bony alignment is necessary. Typically, the center of the hip, knee and ankle are collinear in normal anatomic alignment. This allows the joints and the joint orientation angles to be roughly parallel to the floor. In patients with genu varum or “bowed legs”, the knees are located outside or lateral to a normal mechanical axis, whereas in patients with genu valgum or “knock knees” the knees are located inside or medial to a normal mechanical axis.

**Figure 2:**
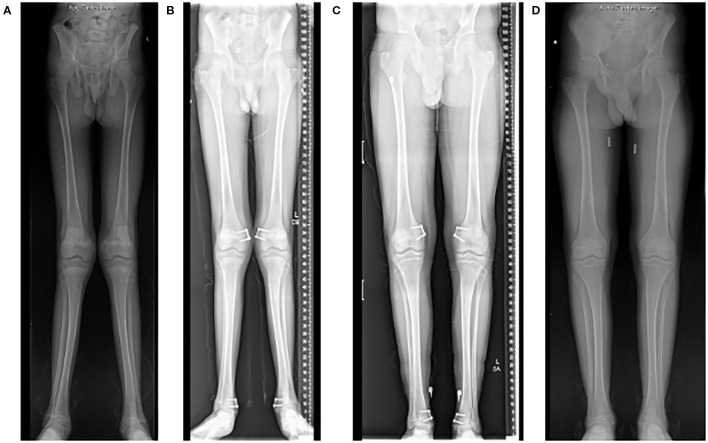
Radiographs of a 15-year-old who presented with significant genu valgum (“knocked knees”) **(A)**. He underwent a guided-growth procedure using tension band plates on the medial side of both his distal femur and distal tibia physes **(B)**. The hardware was removed 2 years after the procedure once satisfactory deformity correction was achieved **(C, D)**.

In skeletally immature patients, guided-growth surgery is preferred, if possible, as it is much less invasive and has a relatively short recovery period, especially compared to formal osteotomies and deformity correction. Guided growth procedures capitalize on the growth of the long bones to correct deformity and prevent further progression. Guided growth can be utilized when the child has at least 2 years of growth remaining in mild to moderate long bone deformities. Guided growth surgery involves a temporary hemiepiphysiodesis using small plates and screws that tether the physis on the convex side of the deformity, i.e. laterally in genu varum and medially in genu valgum. The hardware tethers the convex side of the physis and acts as an extraphyseal fulcrum, allowing the opposite (or concave) side of the physis to continue growing. Once satisfactory deformity correction has been achieved, the hardware is removed to prevent overcorrection and to allow for continued balanced growth (**Figure 2**).

**Figure 3:**
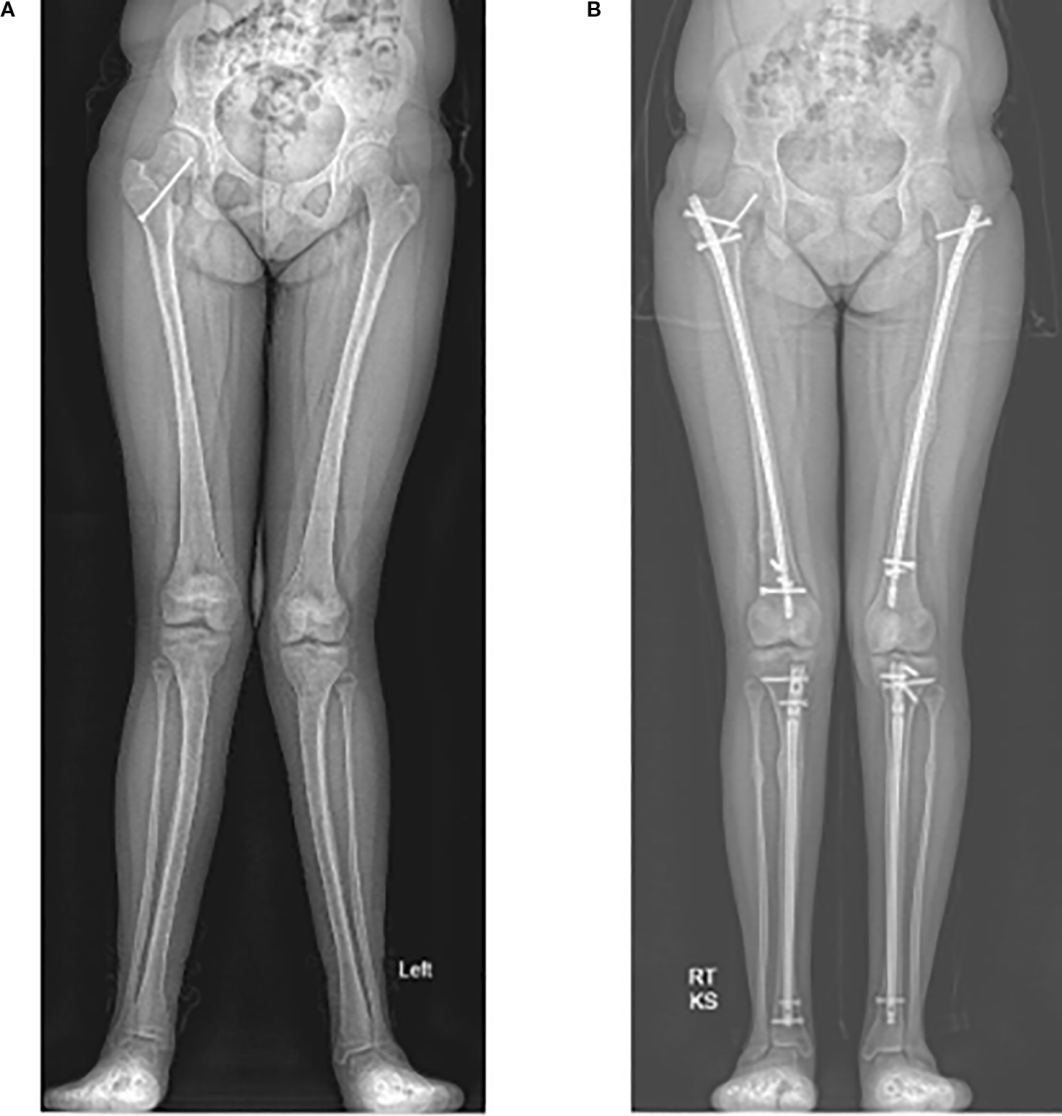
Radiographs of the same patient, who at 17 years of age underwent posterior spine fusion for thoracic-level scoliosis **(A, B)**. Of note, he received a renal transplant at age 16.

If deformity correction is not achieved with guided-growth procedures, the child may be indicated for formal osteotomy and deformity correction (**Figure 3**). Additionally, if the patient presents near or at skeletal maturity, guided-growth procedures are ineffective, and the surgical treatment options are limited to formal osteotomies. Fixation can be done with a plate and screws, an intramedullary nail, or, if gradual correction of the deformity is indicated, an external fixator. Internalized hardware can remain for the duration of the patient’s life or be removed if bothersome, while external fixation will be removed once the deformity is corrected, and the bone has healed.

### Spinal deformity

In patients with cystinosis, it is recommended to follow the general practice guidelines for monitoring and treating scoliosis. If the deformity is less than 50 degrees in a skeletally immature patient, they may be a candidate for customizable bracing and close observation. A rigid orthotic brace is tailor made for the patient’s curve. It typically rests upon the hips and spans the torso up to the shoulder blades, bolstering the concave portion of the curve. These braces are most effective when worn 16–23 hours per day. This conservative treatment is not intended to correct the deformity but rather prevent its progression.

**Figure 4:**
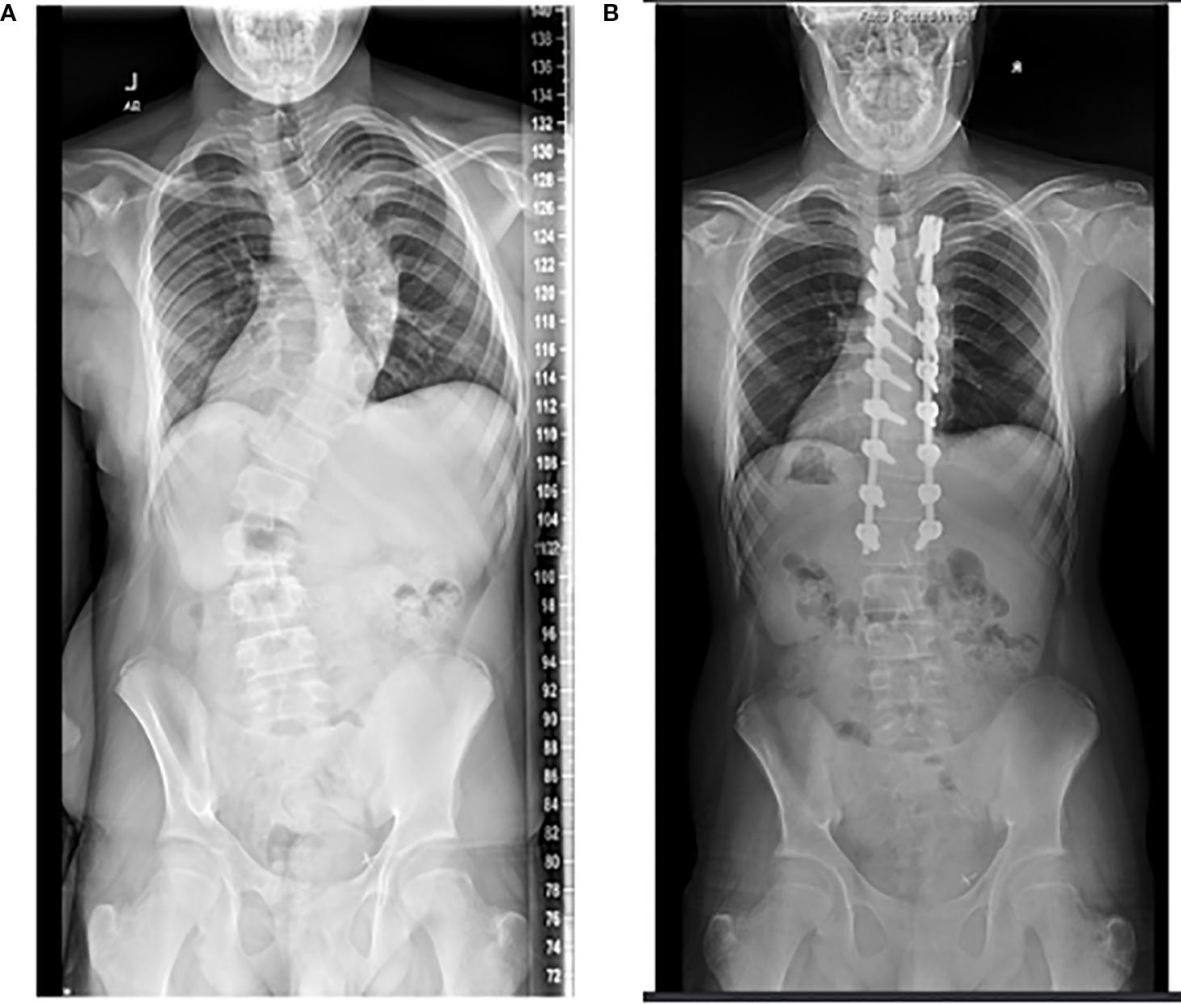
Radiographs of a 12-year-old with significant genu valgum **(A)**. She underwent left sided corrective osteotomies and intramedullary nailing of both the femur and tibia at 13 years old. She then underwent right sided deformity corrections at the age of 14. Radiograph of the corrected lower limb alignment at 15 years old **(B)**.

Spine fusion surgery is usually considered when the angle of deformity is greater than 50 degrees in the e plane. Once the deformity is that large, even if the patient is skeletally mature, it is expected for the curve to continue to progress by 1–2 degrees annually. During surgery, an extensive posterior approach to the spine is performed and two rods are secured to either side of the vertebrae using pedicle screws and/or hooks. As the rods are placed, the typical “S” shaped deformity is corrected in the coronal plane and kyphosis and/or lordosis is re-introduced in the sagittal plane (**Figure 4**).

For patients found to have vertebral insufficiency fractures, either conservative management with a brace or a smaller construct posterior spinal fusion involving the vertebrae above and below the fracture can be used to treat the injury depending on its severity.

## Outcomes

There is little published literature on the long-term outcomes of orthopedic surgery in cystinosis patients in terms of functional recovery, recurrence and complications. If outcomes can be extrapolated from other metabolic bone disorders like X-linked hypophosphatemia, these children are expected to have lower quality of life scores and are at risk factor for further osteoarthritis (1, 22). Additionally, those that undergo renal transplantation and are likely on chronic glucocorticoids or immunosuppressive drugs have increased loss of bone mass and higher risk of fractures (1). Ultimately, focused research is needed to evaluate long-term outcomes of the musculoskeletal manifestations of cystinosis.

## Summary

Although the renal manifestations of cystinosis have been well researched, there remains a paucity of literature regarding the orthopedic ramifications of the disease. The most common issues include lower limb deformities, scoliosis, myopathy, and fragility fractures. Pediatricians should clinically screen for scoliosis and long bone deformities and refer early to a pediatric orthopedic surgeon for monitoring and treatment. Strict medical optimization is necessary prior to surgically addressing orthopedic problems. This includes lowering cystine concentrations throughout the body with cysteamine, a cystine binder, as well as electrolyte and hormonal replacement therapy. Once medically optimized, a skeletally immature patient can be considered for minimally invasive guided growth surgery with temporary hemiepiphysiodesis in the extremities. After skeletal maturity, the deformities may be corrected with formal osteotomies and fixation. Spinal fusion is considered for curves greater than 50 degrees in the coronal plane.
