# Supplementary material for: The assessment and treatment of the musculoskeletal manifestations of cystinosis
Source: Front Nephrol. 2025 Sep 25;5:1624586. doi: 10.3389/fneph.2025.1624586 (PMC12507550; doi:10.3389/fneph.2025.1624586)
Supplement: Supplementary file 1 [file Table1.docx]

Supplemental Table 1. Literature Review and Summary Table of all Major Studies

| Study Name | Journal | Type of Study | Number of Patients | Key MSK Findings | Treatment Outcomes/ Conclusions |
| --- | --- | --- | --- | --- | --- |
| Gahl et al (2002) | NEJM | Review | - | Cystinosis is associated with significant growth retardation, renal osteodystrophy, and rickets | Early diagnosis of cystinosis is paramount for successful treatment. Fluid and electrolyte replacement can prevent progression. Cysteamine depletes lysosomal cystine over the long-term, delaying glomerular failure and improving growth. |
| Zimakas et al (2003) | Pediatr Nephrol | Educational Review | - | Bones X-ray and DEXA scans should be obtained if there is renal osteodystrophy.  Bone marrow biopsy reveals cells packed with cystine crystals  Children with FS exhibit plasma and muscle carnitine deficiency due to failure to reabsorb carnitine  About one-third of adult patients are affected with progressive distal vacuolar myopathy, with severe muscle wasting in some cases (Fig. 2e, f) characterized by type 2 fiber atrophy and cystine accumulation in perimysial cells | Oral phosphate replacement with the addition of Vitamin D for better absorption  Carnitine supplementation can increase plasma carnitine level in NC patients, but clinical benefit has not been demonstrated.  Children benefit from growth hormone, which enhances growth [36] and may also increase phosphate reabsorption [18]; although well-treated patients usually grow well and do not require growth hormone. |
| Besouw et al (2014) | JIMD Reports |  | 5 | At study onset, plasma free carnitine was normal in all patients, total carnitine (1/5), acetylcarnitine (3/5), and several short- and medium-chain acylcarnitines ≤10 carbons (5/5) were increased indicating carnitine over-supplementation. Three months after cessation, carnitine profiles normalized and 3/5 patients showed plasma carnitine deficiency. Three months after reintroduction, plasma free carnitine normalized in all patients, however, carnitine profiles were disturbed in 4/5 patients. Urine free carnitine, acetylcarnitine, and acylcarnitines ≤10 carbons were increased in all patients independent of carnitine supplementation. | Administration of recommended doses L-carnitine (50 mg/kg/day) resulted in over-supplementation. Although the drug is considered to be rather safe, long-term effects of over-supplementation remain unknown warranting cautious use of high doses. Plasma carnitine profile might be used as a monitor, to prevent overdosing. |
| Bacchetta et al (2016) | BoneKEy Rep | Case Reports | 3 | Patient 1 is a girl who was diagnosed with nephropathic cystinosis lately at 5 years of age (clinical picture of renal Fanconi syndrome but delay in diagnosis); from 7 years of age, she developed progressive genu valgum with important bone pains and decreased walking ability, thus requiring surgical correction and bone biopsy at 13 years of age  Patient 2 is a boy diagnosed with cystinosis at 2 years of age; in addition to a neurological developmental delay of unknown etiology, he developed severe bone deformation with a progressive and complete impairment of walking ability from 12 years of age. A surgical correction and bone biopsy were therefore performed at 14 years of age  Patient 3 is a boy from a consanguineous family who was treated with cysteamine starting at 11 months of age. He was born at 35 weeks gestation with a neonatal course complicated by birth asphyxia, respiratory distress syndrome, pneumothorax and seizures. Surgery was performed at 1 month of age to treat cricoid cartilage stenosis; he was diagnosed with hypothyroidism at 10 months of age. He received growth hormone therapy from age 5 to 11. At 10 years of age, he developed leg and joint pains, bilateral knee valgus, severe scoliosis, muscle weakness, neuromotor regression and dark red skin lesions on both elbows without any history of trauma. His abnormal walking pattern persisted because of severe knee deformities, despite maximized phosphate and vitamin D supplementation, requiring orthopedic surgery to realign his lower limbs at 16 years of age. Eventually, because of a rapid bowing of long bone, bone pains and severe osteopenia, but no evidence for bone fracture, he underwent a bone biopsy at 20 years of age. | From our experience of three pediatric patients with cystinosis and severe bone deformations having undergone a thorough biochemical evaluation, as well as a bone biopsy, we conclude that even though copper deficiency, high-doses cysteamine regimens and abnormal thyroid metabolism may worsen the bone picture in cystinosis patients, the exact pathophysiology of such impairment remains to be defined. |
| Elmonem et al (2016) | Orphanet J Rare Dis | Review | - | A distal vacuolar myopathy presenting as progressive distal muscle wasting and weakness, has been observed in about 24 % of renal transplant cystinosis patients. Myopathy affects patients from their second decade of life. In post-transplant cystinotic patients who did not receive long-term cystine-depleting therapy, cystinotic myopathy may cause an extraparenchymal pattern of restrictive lung disease as well as swallowing dysfunction. | Several lines of treatment are available for cystinosis including the cystine depleting agent cysteamine, renal replacement therapy, hormonal therapy and others; however, no curative treatment is yet available. |
| Claramunt et al (2018) | Nephrol Dial Transplant | In vitro | - | CMBD is related to cystinosin-induced osteoclastic dysfunction | Low doses of cysteaine may partially correct the CTNS-induced osteoclast dysfunction in NC, and stimulate osteoblastic differentiation and mineralization. High doses inhibits these functions. |
| Bertholet et al (2018) | Pediatr Nephrol | Prospective Cohort Study | 10 | Patients with nephropathic cystinosis display significant clinical bone symptoms  Patients with nephropathic cystinosis display significant cortical impairment as assessed with HR-pQCT | Bone impairment (rather cortical than trabecular) in nephrogenic cystinosis corresponds to a real clinical problem; indeed, in this pilot study, 70% of patients displayed significant bone symptoms, during teenage or young adulthood |
| Hohenfellner et al (2019) | J Inherit Metab Dis | Consensus Statement | - | CMBD is associated with bone pain, fractures, skeletal deformities, growth retardation, scoliosis, osteopenia or osteoporosis, progressive myopathy, and sarcopenia. | This consensus is the first of its kind on NC, and established standardized guidelines for recognizing, assessing, managing CMBD. CMBD must be recognized early, treated with individualized care, and followed throughout the patients lifetime to optimize outcomes. Treatment involves medical management, orthopedic intervention, physical therapy, and nutritional support. |
| Florezano et al (2020) | J Am Soc Nephrol | Prospective Cohort Study | 30 | Mean bone mineral density (BMD) Z-scores were decreased in the femoral neck, total hip, and 1/3 radius (p < 0.05)  27% of subjects reported one or more long bone fractures.  32% of subjects had incidental vertebral fractures, which were unrelated to transplant status. Long-bone deformity/bowing was present in 64%; 50% had scoliosis.  Risk factors included CKD, phosphate wasting, hypercalciuria, secondary hyperparathyroidism, hypovitaminosis D, male hypogonadism, metabolic acidosis, and glucocorticoid/immunosuppressive therapy | Skeletal deformities, decreased bone mass, and vertebral fractures are common and relevant complications of nephropathic cystinosis, even before renal transplantation. Efforts to minimize risk factors for skeletal disease include optimizing mineral metabolism and hormonal status, combined with monitoring for nephrocalcinosis/nephrolithiasis. |
| Machucua-Gayet et al (2020) | Int J Mol Sci | Review | - | Established the concept of Cystinosis Metabolic Bone Disease, and that it is more complex than other renal osteodystrophies. Identified five contributing factors:   1. Long-term fanconi syndrome effects 2. Nutritional deficiencies 3. Hormone imbalances 4. Myopathy 5. Intrinsic bone lesions | CMBD is unique and complex, further work important for better understanding |
| Ewert et al. (2020) | J CLin Endocrinol Metab | Cross-sectional multicenter study | 129 | 11x greater risk of MSK comorbidity in nephropathic cystinosis (NC) than CKD | NC is associated with more significantly defective bone metabolism than CKD. only partially normalizes after transplant |
| Iyob-Tessema et al. (2021) | Kidney Int Rep | Cross-sectional observational study | 76 | Significantly weaker muscle strength in patients with cystinosis when compared with CKD, even more so in men and late initiation of cysteamine | Further research needs to be done on the association of physical therapy and cysteamine therapy timing to optimally treat CMBD |
